# Thirty years of volcano geodesy from space at Campi Flegrei caldera (Italy)

**DOI:** 10.1038/s41597-022-01849-7

**Published:** 2022-11-26

**Authors:** Marco Polcari, Sven Borgstrom, Carlo Del Gaudio, Prospero De Martino, Ciro Ricco, Valeria Siniscalchi, Elisa Trasatti

**Affiliations:** Istituto Nazionale di Geofisica e Vulcanologia (INGV), Via di Vigna Murata 605, 00143 Rome, Italy

**Keywords:** Volcanology, Natural hazards, Research data

## Abstract

This work provides the mean ground deformation rates and ground displacement time series of the Campi Flegrei caldera (Italy) retrieved by satellite remote sensing data analysis from 1992 to 2021. Synthetic Aperture Radar (SAR) images acquired by ERS 1–2 (1992–2002), Envisat (2003–2011) and Cosmo-SkyMed (2011–2021) are processed by multi-temporal SAR Interferometry (InSAR) approach using the same technique, parameters and reference system, to obtain for the first time a homogeneous and time-continuous dataset. The validation of the InSAR products is carried out by comparison with the measurements provided by precise levelling lines and cGNSS stations. The produced outcomes offer an overview on the temporal behaviour of ground deformation at Campi Flegrei along an unprecedented time window of about 30 years and can be exploited by the scientific community for supporting and improving the knowledge of the dynamics of the caldera.

## Abstract (summary)

**Table:** 

Measurement(s)	ground deformation process
Technology Type(s)	satellite imaging of a planet
Factor Type(s)	Satellite Line-of-Sight Displacement
Sample Characteristic - Environment	ground deformation process
Sample Characteristic - Location	Campi Flegrei

## Background & Summary

Campi Flegrei is a volcanic caldera located in southern Italy, west of the city of Naples, well known by the scientific community because of the very high volcanic risk associated. It is indeed a highly urbanized area undergoing periodic phases of unrest, causing inflation or deflation with ground deformation rate up to some mm/month and several related effects such as shallow depth seismic swarms^1,2^, soil temperature variations and degassing in the caldera center, i.e in the Solfatara-Pisciarelli volcanic district^3–5^. Such phenomenon of ground displacement, known as the *Campi Flegrei bradyseism*, has also been mapped by archaeological records^6,7^. It is directly connected to the volcanic activity and can be exploited to retrieve information about the source geometry and its depth thus providing important indications for hazard assessment and risk mitigation purposes^8–11^.

There are several techniques able to detect ground movements, such as the continuous Global Navigation Satellite System (cGNSS) and the levelling surveys. cGNSS provides measurements with high temporal sampling of any movements interesting the ground station along the 3D displacement component, i.e. North-South (NS), East-West (EW) and Vertical (UP). Instead, the levelling technique measures the altitude of the benchmarks constraining the vertical changes of the quote in the time interval between two measurement campaigns^12^. Then, they are both very useful to retrieve precise information about the Campi Flegrei ground displacement but only in a limited number of ground-based stations. In order to have a synoptic view of the deformation phenomena affecting Campi Flegrei caldera, satellite data acquired by Synthetic Aperture Radar (SAR) sensors can be used. Indeed, SAR images processed by multi-temporal SAR Interferometry (InSAR) approaches allow to obtain a very huge number of point targets and thus to detect ground deformations of Campi Flegrei caldera with dense spatial coverage. Since 1992 with the launch of the first space mission equipped with a SAR sensor operating for more than one year, the ERS 1–2 missions of the European Space Agency (ESA), InSAR data have been largely applied in the study of Campi Flegrei with particular focus on the intense inflation phase started in 2011 and still ongoing^13–16^.

Actually, there are several platforms and services aiming at providing software for data processing and databases of InSAR products such as the GEP - Geohazards Exploitation Platform (https://geohazards-tep.eu/#!) and the COMET-LiCS InSAR portal^17^ which work with Sentinel-1 data. However, InSAR outcomes retrieved from different data processing centers could be affected by slight differences depending on the applied technique, processing parameters, pixel spacing and most of all the reference system. Moreover, only limited time intervals have been analysed according to the aim of specific studies or in response to the institutional stakeholders requests (e.g. the Civil Protection Agencies). This produces diffused data fragmentation with large amounts of InSAR data that are therefore not fully exploited in their capability for quantitative and time continuous ground deformation monitoring and for supporting the advancement of knowledge related to the Campi Flegrei dynamics.

**Fig. 1:**
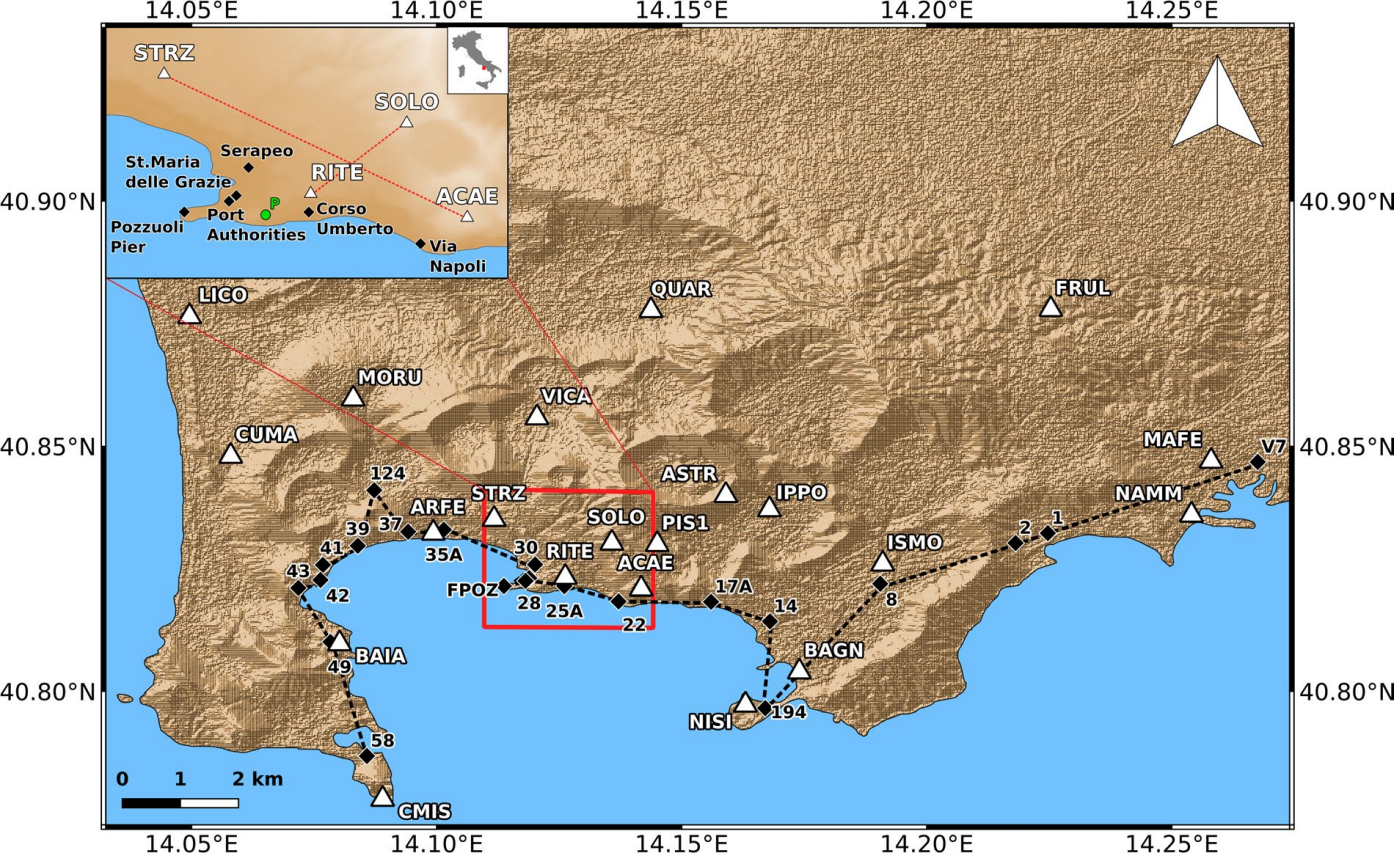
Focus on the Campi Flegrei (Italy) area. The white triangles indicate the cGNSS stations belonging to the NeVoCGNSS network. The black diamonds are the levelling benchmarks along the line crossing, from west to east, the Miseno-Pozzuoli-Napoli coastline. The inset shows the inner area of the caldera, affected by the most intense ground displacements. The green point P in the inset is the InSAR target showing the maximum vertical deformation. The background image is the 12 m TanDEM-X DEM.

Then, the goal of this work is to provide a homogeneous and continuous InSAR dataset comprehensive of SAR images acquired since the first space mission to recent ones, freely accessible by the entire scientific community and available to be exploited for future studies about Campi Flegrei caldera (Fig. 1).

To this aim, ERS 1-2 data from 1992 to 2002, Envisat data from 2003 to 2010 and Cosmo-SkyMed (CSK) data from 2011 to 2021 were processed by the GeoSAR Laboratory of INGV, an infrastructure specifically designed for SAR data processing, interpretation and modelling, using the same technique, parameters and reference system. The retrieved ground velocity maps and displacement time series offer an overview on the temporal behaviour of Campi Flegrei ground displacement along an unprecedented time window of about 30 years. Additionally, the validation of the InSAR products are provided by comparison with the measurements provided by precise levelling lines and cGNSS stations belonging to the Neapolitan Volcanoes Continuous cGNSS (NeVoCGNSS) network.

## Methods

The remote sensing dataset used for providing the outcomes of the present work consists of SAR images acquired from ERS 1–2, Envisat and CSK space missions from 1992 to 2021 along ascending and descending tracks. The acquisition geometry is quite similar for ERS 1–2 and Envisat missions**:** the angle between the incident radar beam and the direction perpendicular to the ground surface, i.e. the incidence angle, spans from about 21° and 23°, whereas the clockwise angle between the north and the orbit direction, i.e. the azimuth angle, is ~14° for both tracks. Instead, CSK missions acquire with incidence angle of ~49° for ascending and ~26° for descending track and with an azimuth angle of ~13° for both. In Table 1 the characteristics of each SAR dataset are summarized.

**Table 1:** Summary of the characteristics of the dataset produced in this work.

Mission	Track	Time Span	Incidence Angle	Azimuth Angle	Number of images	Range × Azimuth Looks
ERS 1–2	Ascending	1993–2002	23°	−14°	47	2 × 10
ERS 1–2	Descending	1992–2001	21°	14°	58	2 × 10
Envisat	Ascending	2002–2010	23°	−14°	62	2 × 10
Envisat	Descending	2003–2010	21°	14°	56	2 × 10
CSK	Ascending	2011–2021	49°	−13°	603	15 × 15
CSK	Descending	2012–2021	26°	13°	127	15 × 15

**Fig. 2:**
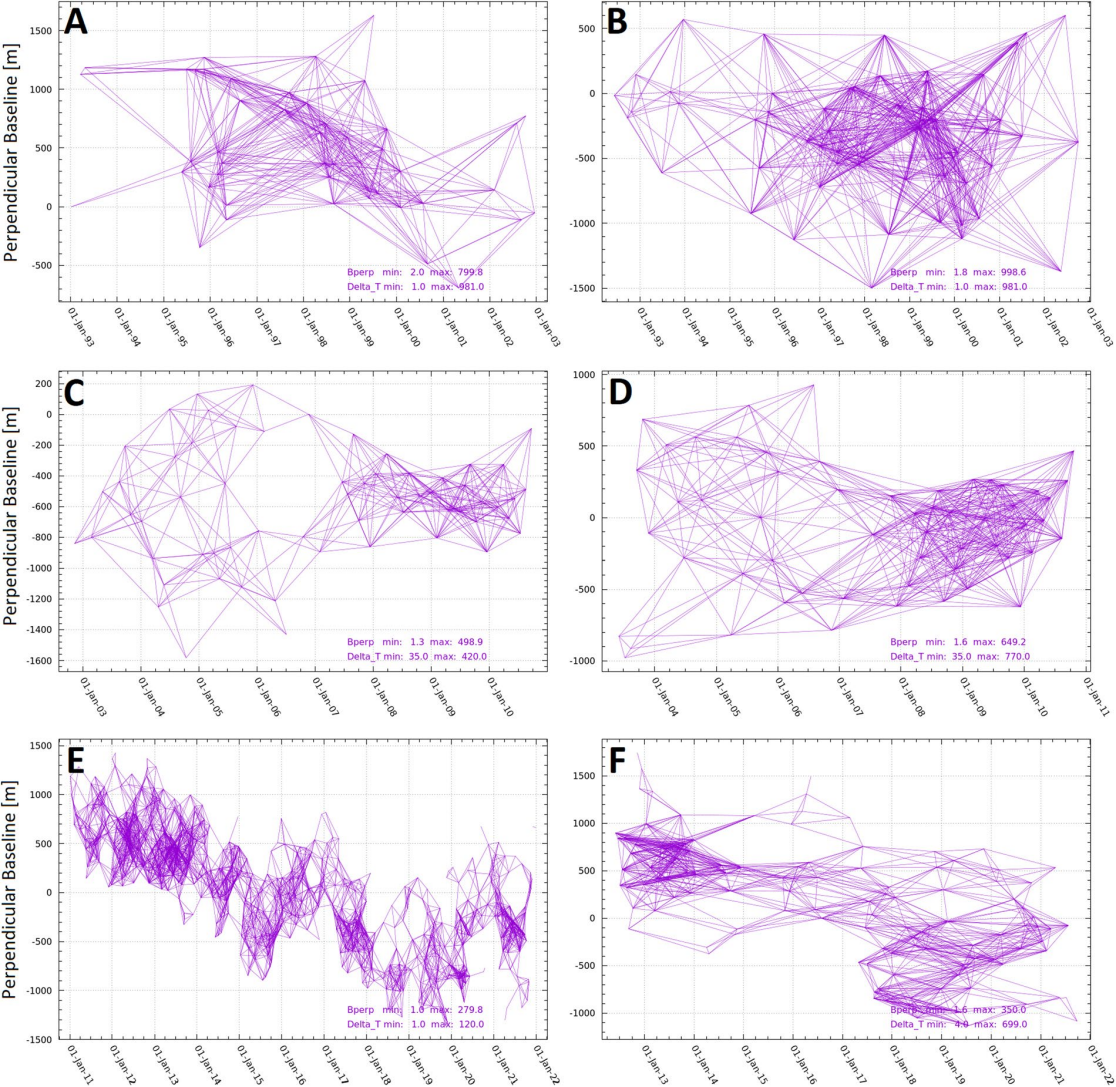
Interferograms networks retrieved with ERS Ascending (**A**) and descending (**B**) data, Envisat ascending (**C**) and descending (**D**) data and CSK ascending (**E**) and descending (**F**) data.

InSAR analysis was performed by the multi-temporal/multi-baseline approach^18^ developed in the framework of GAMMA software^19^. Such approach merges the characteristics of the two main advanced InSAR techniques, i.e. Persistent Scatterers (PS)^20^ and Small BAseline Subsets (SBAS)^21^. Then, the interferogram networks were retrieved by setting a maximum perpendicular baseline and temporal gap between pairs of SAR images. In particular, considering the different orbital tube and temporal sampling of the space missions, such thresholds were set to 800 m–1000 m and 1000 days for ERS 1–2 ascending and descending data, to 500 m–650 m and 450–800 days for Envisat ascending and descending data and to 280 m–350 m and 120–700 days for CSK data. These loose thresholds were chosen to obtain dense and redundant interferogram networks such to allow discarding all the interferograms affected by severe errors without losing any connection of the networks (Fig. 2). All SAR images were first averaged to reduce the SAR speckle noise by applying multi-look factors in order to get approximately the same ground pixel spacing in output set to 30 m. Then, based on the retrieved networks, the differential interferograms were estimated and the topographic contribution was removed by means of the 12 m Digital Elevation Model (DEM) acquired by the TanDEM-X mission and delivered in response to the Deutsches Zentrum für Luft- und Raumfahrt e.V. (DLR) Announcement of Opportunity for TanDEM-X products request. Such interferograms were then filtered by Goldstein filtering^22^ setting the window size to 32 pixels and the exponent factor to 0.6 and unwrapped by the minimum cost flow^23^ algorithm using 0.35 as coherence threshold.

Hence, were generated the InSAR point targets maps by selecting the points with temporal coherence greater than values spanning from 0.35 to 0.5 according to the temporal sampling of each mission. It returned a very huge number of points for all the missions, especially for X-band CSK data, guaranteeing a dense spatial coverage of all the Campi Flegrei caldera. Such point targets maps were used to sample the interferograms thus moving from 2D Raster to a much lighter, flexible e manageable vector format. The final solution in terms of displacement time series and linear velocity was then found by an extension of the Singular Value Decomposition (SVD) based Least-Squares inversion^21^. In particular, for each point target, this algorithm considers a set of linear equations as a function of the deformation velocities along the considered time intervals. Such matrices are augmented with a set of additional weighted constraints on the ground acceleration, to emphasise or penalise any fast velocity variations with respect to the linear trend. These constraints are tuned by a weighting factor, **γ**, which can be set to a value from 0 to retrieve a no-smoothed solution to >10 for quasi-linear solutions. Considering that the Campi Flegrei bradyseism is characterised by several ground acceleration/deceleration phases, the weighting factor **γ** was set to 1.5 to take into account also non-linear deformation components.

The point target chosen as reference point for all the InSAR measurements is located in the proximity of the cGNSS AGR1 station in Portici. It is placed on the rooftop of the Faculty of Agriculture of University of Naples Federico II and showed a very stable behaviour during a long time span with velocity values close to zero^24^.

## Data Records

The produced dataset consists of a shapefile for each of the 3 space missions and for the ascending and descending tracks, thus a total of 6 shapefiles. They are located in a data repository managed by INGV and can be freely accessed via the following Digital Object Identifier: 10.13127/insar/ts^25^. This open the page of all the InSAR products performed by INGV GeoSAR Laboratory and InSAR working group. To download the data shown in this work, the users can go to *download data* tab and search for Campi_Flegrei subfolder under the *Volcanoes* folder, otherwise they can be directly downloaded via the following link: http://www.geosar-iridium.ct.ingv.it/landing/tmp/download.php?dir=/home/data/iridium/GeoSAR_INGV_Archive/InSAR_ground_displacement_time_series/Volcanoes/Campi_Flegrei&folder=Campi_Flegrei.

Each shapefile is a table collecting the InSAR point targets with the columns representing the following attributes for each point: Latitude, Longitude, Height, Deformation velocity expressed in mm/yr, Velocity error expressed in mm/yr and Line-of-Sight (LoS) Displacement in mm for every date.

The files are named with an encoding of 7 fixed-length fields, where:

- the first field identifies the kind of product (TS is Time Series);
- the second field identifies the provider of the product (INGV);
- the third field identifies the country where the studied area is located according to the standard ISO3166-1 alpha-3 (https://www.rallybel.com/it/links_iso_code3.html);
- the fourth field identifies the name of the SAR sensor (ERS, ENV for Envisat and CSK for Cosmo-SkyMed);
- the fifth and the sixth fields represent the year of the first and the last image;
- the seventh field is a random code used to uniquely identify the product.

As an example, the shapefile of InSAR results related to CSK ascending data is named TS_INGV_ITA_CSK_2011_2021_GHT. Moreover, each file is provided with two metadata files (.xml), according to INGV (TS_INGV…) and EPOS (Dts_INGV…) standards, in compliance to European guidelines, that summarize the main parameters related to the SAR acquisitions and the InSAR processing, such to guarantee the data reproducibility. All the provided shapefiles can be easily managed in any GIS environment and they are already formatted to be compatible with most of the QGIS plugin, including the tools for analysis and visualization of time series.

**Fig. 3:**
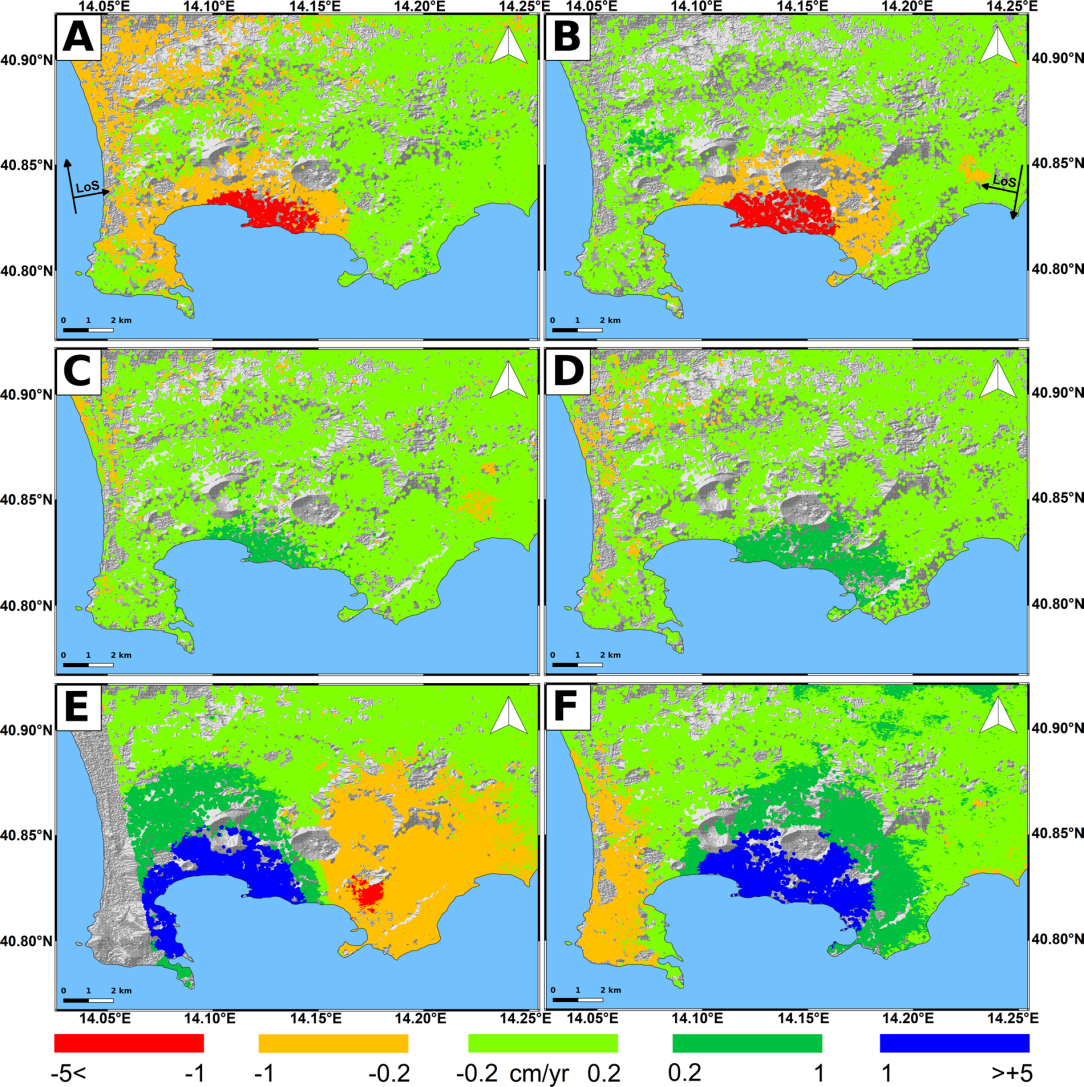
Campi Flegrei LoS deformation rates retrieved from: ERS 1–2 ascending (**A**) and descending (**B**) data during 1992–2002; Envisat ascending (**C**) and descending (**D**) data during 2003–2010; CSK ascending (**E**) and descending (**F**) data during 2011–2021. The background image is the 12 m TanDEM-X DEM.

The retrieved InSAR products in terms of ground deformation rate at Campi Flegrei are shown in Fig. 3. The retrieved measurements are the displacement fields along the LoS of each satellite which is the combination of UP and EW deformation, being the SAR sensors poorly sensitive to NS movements. The first period (1992–2002) is characterized by a subsidence of the central part of the caldera since both orbits show negative velocities up to −5 cm/yr. During the second time window (2003–2010) the mean velocities are slightly positive (values up to 1 cm/yr), while in the last period (2011–2021) they increase to more than 5 cm/yr maximum, affecting mostly the center of the caldera.

The availability of data along both tracks allows to compute the UP and EW velocities, although introducing further errors. Then such analysis was additionally performed, firstly interpolating the ascending and descending LoS velocities on a grid of about 60 m and then estimating the two components for each space mission according to the following equations^26^:

$${V}_{UP}=\frac{{V}_{DSC}\sin {\theta }_{ASC}+{V}_{ASC}\sin {\theta }_{DSC}}{\sin \left({\theta }_{DSC}+{\theta }_{ASC}\right)}$$

$${V}_{EW}=\frac{{V}_{DSC}\cos {\theta }_{ASC}-{V}_{ASC}\cos {\theta }_{DSC}}{\sin \left({\theta }_{DSC}+{\theta }_{ASC}\right)}$$

where:

- *V*_*UP*_, *V*_*EW*_ are the velocities along the vertical and horizontal (east-west) components;
- *V*_*DSC*_, *V*_*ASC*_ are the InSAR descending - ascending velocities along the satellite LoS;
- *θ*_*DSC*_, *θ*_*ASC*_ are the incidence angles for the descending - ascending geometry.

**Fig. 4:**
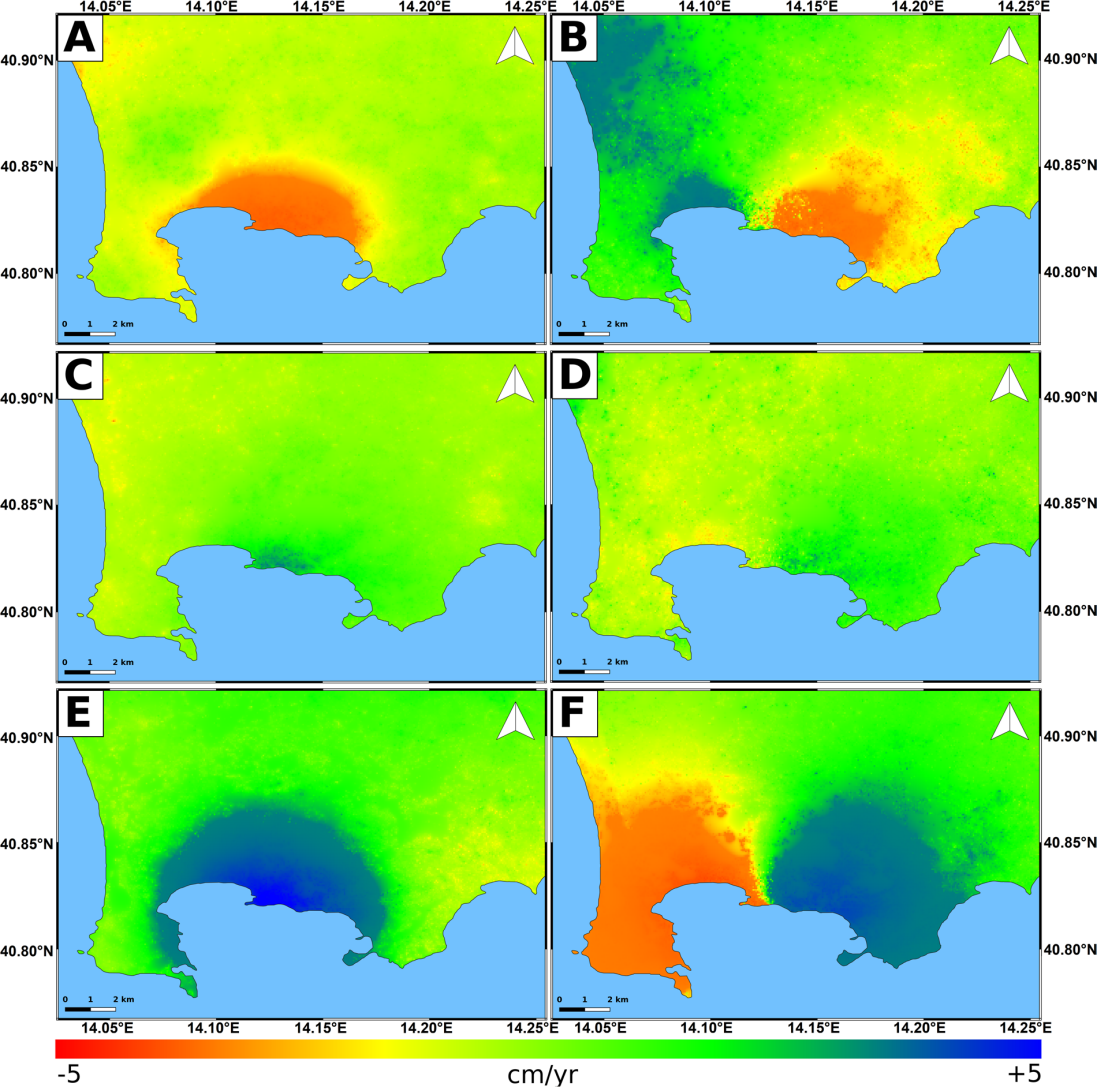
Campi Flegrei UP and EW deformation rate retrieved from: ERS 1–2 data (**A**,**B**) during 1992–2002; Envisat data (**C,D**) during 2003–2010; CSK data (**E,F**) during 2011–2021.

The results are shown in Fig. 4 confirming the change from deflation pattern (A and B panels) to low-rate inflation (C and D panels) and, finally, to a marked inflation (E and F panels).

**Fig. 5:**
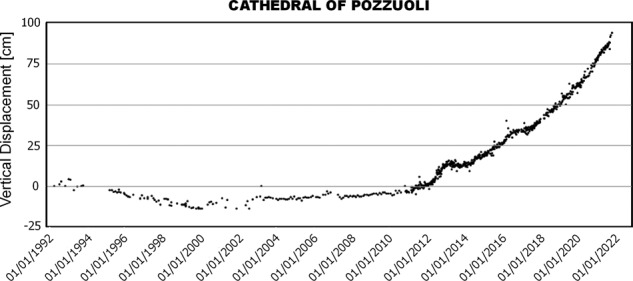
Vertical time series retrieved by combining the ascending and descending data of all the the space missions at point target P shown in Fig. 1 (green circle in the inset) located in Rione Terra in Pozzuoli.

In the following section are reported the LoS displacement at some InSAR point targets, with their validation against independent data. Instead, vertical and horizontal time series cannot be estimated for all the points due to the different spatial coverage and temporal sampling of ascending and descending measurements for the sensors considered, especially for the CSK missions. Only where the deformation is almost vertical is possible to move from LoS to UP time series, since the temporal gaps between orbits can be filled by projecting only one of the two. Accordingly, Fig. 5 shows the vertical displacement time series of the green point *P* in the inset of Fig. 1 located in Rione Terra (Pozzuoli) area, specifically in the proximity of the Cathedral of San Procolo Martire, where the maximum deformation is detected. Indeed, here the vertical deformation is predominant with respect the horizontal one, as also shown for the cGNSS RITE station in De Martino *et al*.^24^.

## Technical Validation

To validate the satellite InSAR dataset, ground measurements retrieved by both levelling surveys and the cGNSS network were exploited.

Vertical ground deformations have been monitored by periodic measurement campaigns along a first levelling network established in 1905 and later on implemented up to 30 benchmarks by the Istituto Geografico Militare. Between 1970 and 1972, levelling measurements were carried out by the latter and by Ministero dei Lavori Pubblici. In 1975, the Osservatorio Vesuviano established a dense altimetric network, including the benchmarks of the preexisting network still available.

Nowadays the high precision levelling includes 370 benchmarks for a total of about 150 km of levelling line with 14 linked loops. The relative measurements have always been subjected to least squares compensation because such a configuration permits minimising and checking the errors associated with altimetric measurements^12^.

Concerning these data, the levelling line crossing the coastline from Capo Miseno (benchmark 58) to the city center of Naples (benchmark V7) was considered. Such dataset consists of 22 benchmarks installed as early as 1985 and whose measurements have been retrieved in several surveys carried out from 1992 to 2018. In order to compare the levelling measurements with the InSAR ones, the vertical ground velocity was estimated along three different time intervals approximately matching the time spans covered by the space missions, i.e. 1992–2002, 2002–2010, and 2011–2018 which is the date of the last campaign (Table 2). At the same time, the measurements of the InSAR point targets surrounding each benchmark within a circle of 50 m radius were averaged. Then, for such averaged measurements were computed the InSAR vertical velocity by combining ascending and descending deformation rates.

**Table 2:** Levelling dataset used for the technical validation of InSAR products.

Benchmark	Location	X	Y	Velocity [1992–2002]	Velocity [2002–2010]	Velocity [2011–2018]
14	Bagnoli	429831	4518474	−4, 9	1, 3	16
17 A	La Pietra	428822	4518927	−12, 7	2	32, 1
22	Via Napoli	427231	4518951	−25, 8	3, 7	59, 5
**25 A**	Corso Umberto	426301	4519308	−27	3, 8	63, 4
28	St. Maria Grazie	425698	4519496	−26, 1	1, 8	56, 4
FPOZ	Molo	425261	4519320	−23, 7	2, 2	—
30	Serapeo	425803	4519803	−25, 3	2, 5	57
35 A	Ripa Puteolana	424241	4520607	−13, 1	−0, 2	27, 7
37	Portus Julius	423627	4520568	−10, 1	−1	18, 8
39	Lucrino 1	422758	4520259	−7	−2, 6	9, 4
41	Lucrino	422153	4519831	−5	−2, 1	5, 5
42	Punta Epitaffio	422108	4519491	−4, 6	−2, 3	5, 4
43	Baia	421725	4519312	−2, 7	29, 2	−
49	Baia castle	422265	4518084	−0, 5	−2, 2	8, 4
58	Miseno	422865	4515494	−0, 9	−1, 7	—
MPOZ	Capitaneria porto	425636	4519434	−25, 1	2, 7	—
194	Molo Nisida	429732	4516510	−2	—	—
124	Apollo Temple	423054	4521512	−4, 4	—	—
V7	Napoli Carmine	438254	4522012	0	−3, 1	—
1	Mergellina	434630	4520430	0	0, 000	−0, 4
2	Tomba Virgilio	434073	4520211	0, 2	0, 000	−0, 1
8	Via Diocleziano	431737	4519310	−1, 3	−0, 1	2,5

The vertical velocity values are expressed in mm/yr. The coordinates (X and Y) of the location of each benchmark are expressed in UTM-WGS84 (zone 33), in meters.

**Fig. 6:**
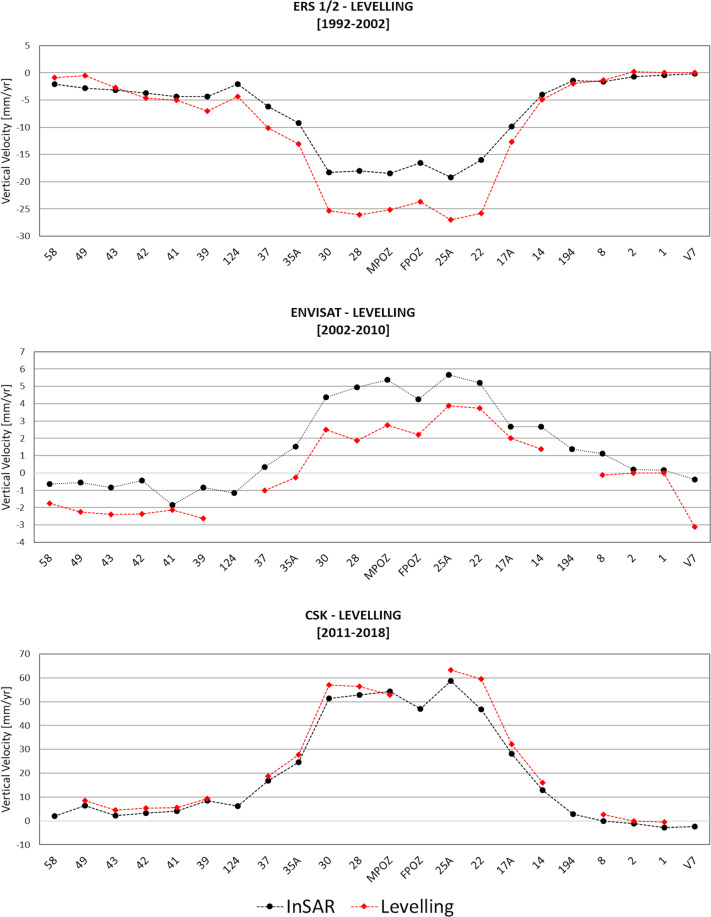
Comparison between ground velocities retrieved from InSAR and levelling data during the three time intervals covered by ERS 1–2 (top panel), Envisat (middle panel) and CSK (bottom panel) space missions.

The result of the comparison between InSAR and levelling data is shown in Fig. 6. It highlights an excellent agreement between the measurements of CSK and Envisat data. The slow (2002–2010) and fast (2011–2018) inflation phase of Campi Flegrei, peaking at the benchmarks located at Via Napoli (benchmark 22), Corso Umberto (benchmark 25 A), the Port Authorities (benchmark MPOZ) and the Pier of Pozzuoli (benchmark FPOZ), the Santa Maria delle Grazie church (benchmark 28) and the Serapeo *(Serapis Temple* - benchmark 30), is well constrained, with differences of few mm/yr comparing the magnitude of the deformation and also the surroundings follow the same behaviour. On the other hand, during the 1992–2002 time interval, the agreement between ERS 1–2 InSAR and levelling data is very good from Miseno (benchmark 58) to Ripa Puteolana (benchmark 35 A) and from La Pietra (benchmark 17 A) to Naples-Piazza del Carmine (benchmark V7). In the area of maximum deformation (from benchmarks 30 to 22), there is instead a shift of about 5 mm/yr between the ground velocities estimated by InSAR and levelling data, although the behaviour is very similar. Such misfit can be due to the intrinsic errors of both techniques which typically are in the order of mm, the difference reference systems, the projection of InSAR ascending and descending rates into the vertical one or a combination of all of them. In any case, excluding the spikes, the Root Mean Square Error (RMSE) between InSAR and levelling measurements is below 5 mm/yr for each mission (Table 3) which is an optimal value considering all the possible error sources.

**Table 3:** RMSE between InSAR and Levelling measurements.

Mission	RMSE [mm/yr]
ERS 1–2	4.42
Envisat	1.72
CSK	4.13

cGNSS measurements from the station belonging to the NeVoCGNSS network span from 2000 to 2019. Due to the lack of cGNSS data before 2000, only the validation with Envisat (2003–2010) and CSK (2011–2021) is computed. De Martino *et al*.^24^ show that the AGR1 cGNSS station is not affected by significant deformations (velocity less than 1 mm/yr), then it can be assumed as the reference for both techniques thus allowing to directly compare InSAR and cGNSS measurements. In order to validate the InSAR dataset, the cGNSS measurements were first projected along the ascending and descending LoS of each satellite according to the following equation:

$${D}_{LoS}={D}_{UP}\cos \theta +{D}_{NS}\sin \theta \sin \alpha -{D}_{EW}\sin \theta \cos \alpha $$

Where:

- *D*_*LoS*_ = Ground displacement projected along the satellite Line-of-Sight
- *D*_*UP*_ = Ground displacement along the vertical component
- *D*_*NS*_ = Ground displacement along the North-South component
- *D*_*EW*_ = Ground displacement along the East-West component
- *θ* = Incidence Angle
- *α* = Azimuth angle

**Fig. 7:**
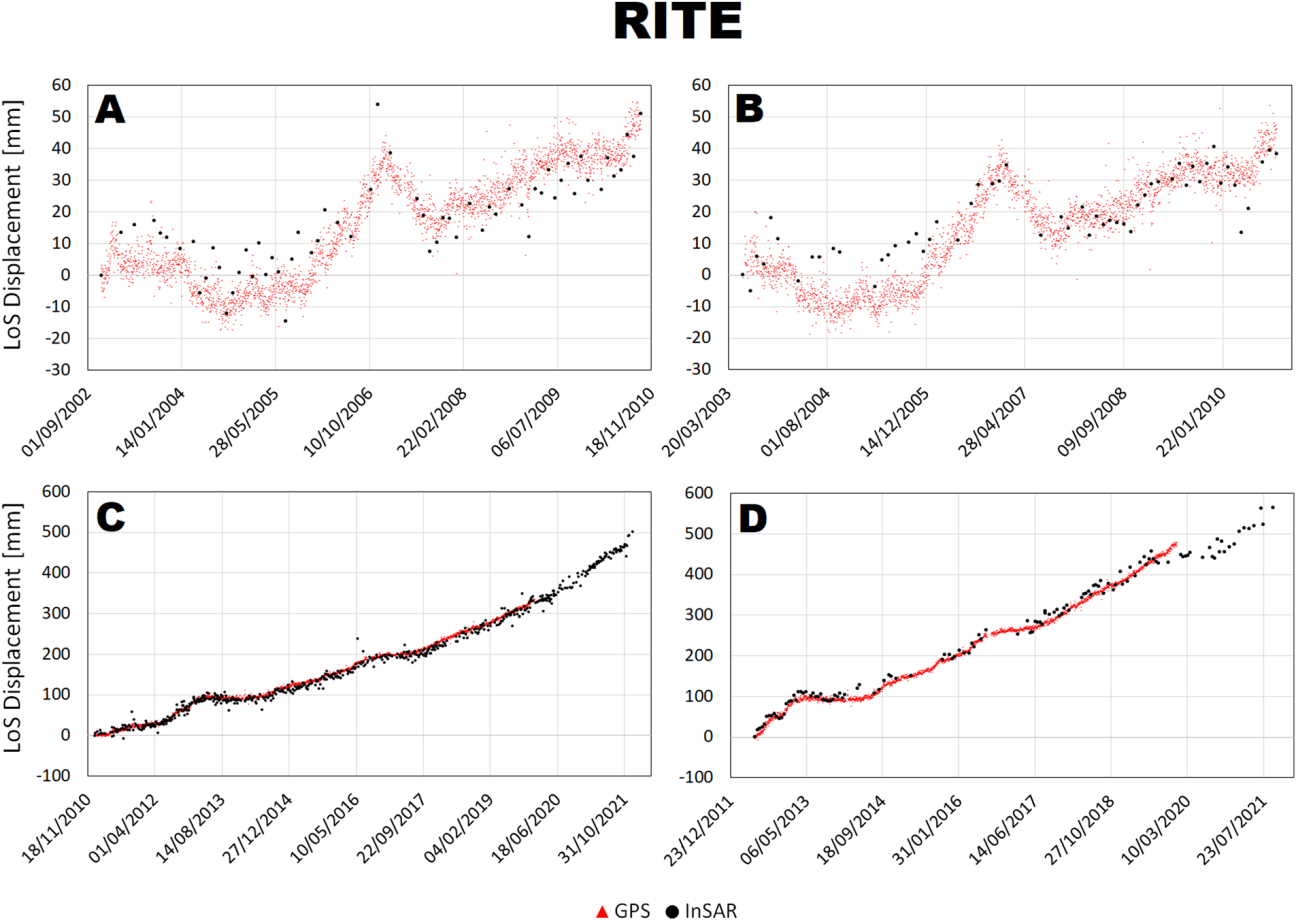
Comparison between InSAR and cGNSS LoS displacement time series at RITE station for Envisat ascending (**A**) and descending (**B**) and CSK ascending (**C**) and descending (**D**) space missions.

**Fig. 8:**
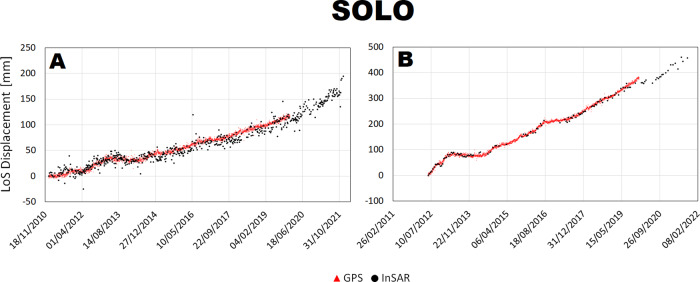
Comparison between InSAR and cGNSS LoS displacement time series at SOLO station for CSK ascending (**A**) and descending (**B**) space missions.

**Fig. 9:**
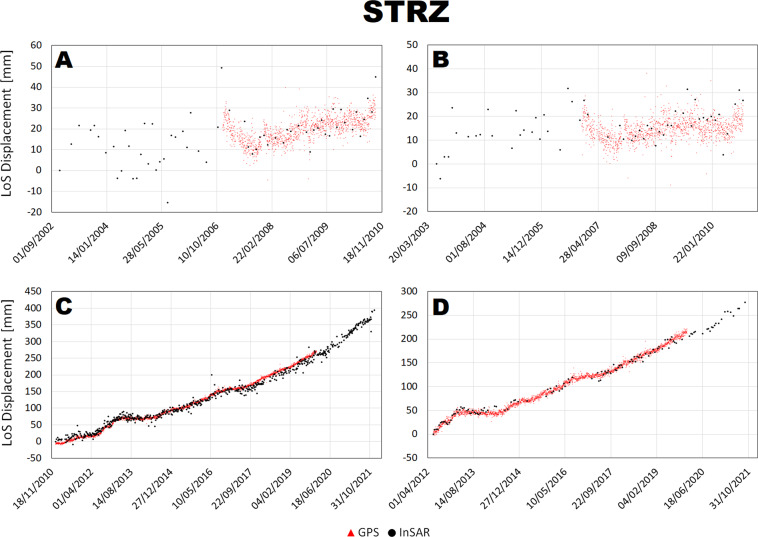
Comparison between InSAR and cGNSS LoS displacement time series at STRZ station for Envisat ascending (**A**) and descending (**B**) and CSK ascending (**C**) and descending (**D**) space missions.

**Fig. 10:**
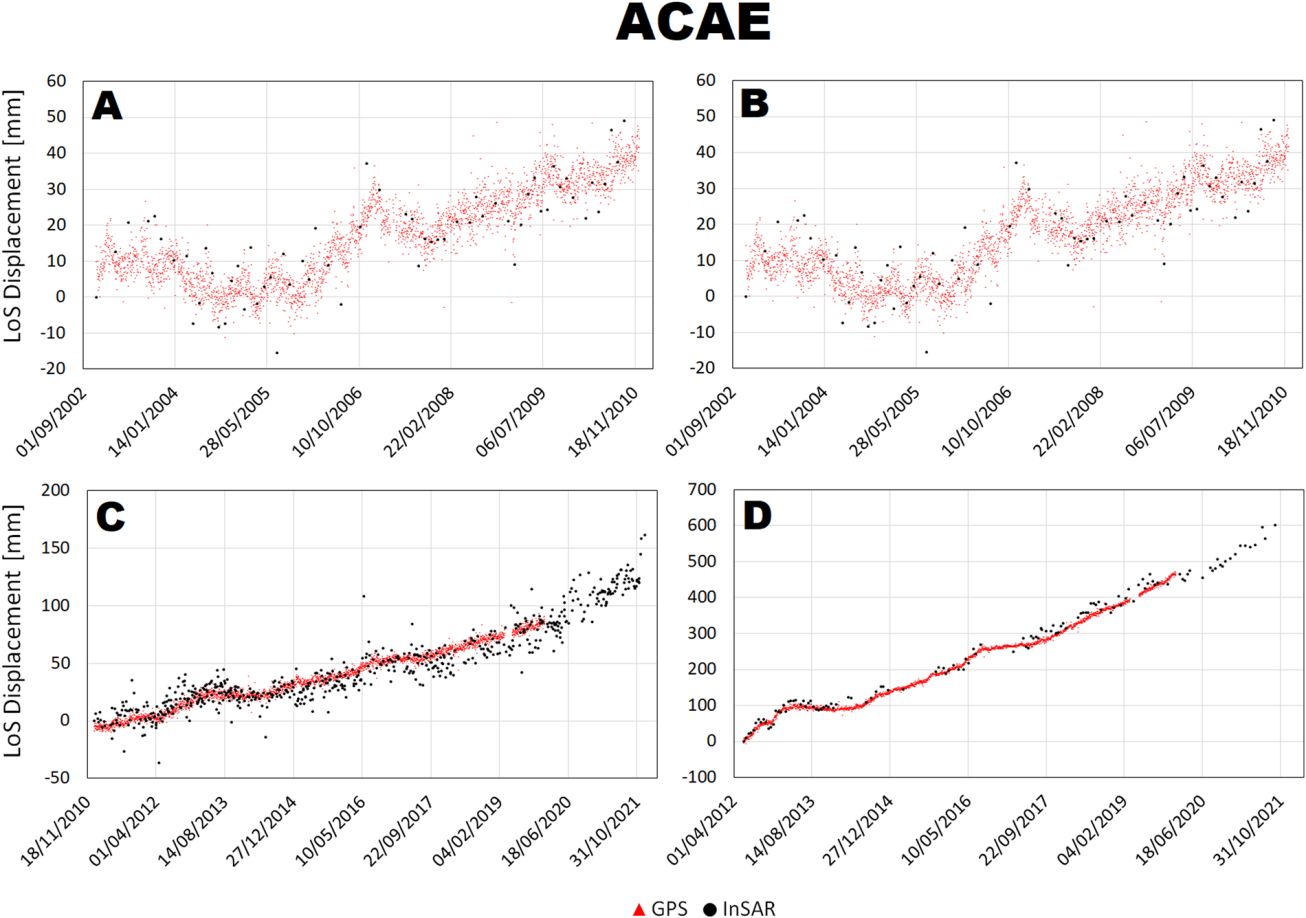
Comparison between InSAR and cGNSS LoS displacement time series at ACAE station for Envisat ascending (**A**) and descending (**B**) and CSK ascending (**C**) and descending (**D**) space missions.

The LoS displacement time series were compared at 4 stations along two transects shown in the inset of Fig. 1, crossing the maximum deformation area NW-SE and SW-NE and connecting the station STRZ-ACAE and RITE-SOLO. Such analysis is shown in Figs. 7–10.

There is a general agreement between InSAR and cGNSS time series for all the 4 stations. Unfortunately, the cGNSS measurements for the stations SOLO and partially for STRZ are missing during the Envisat time window. However, for both RITE and ACAE stations, InSAR and cGNSS time series are consistent along the 2003–2010 time window both showing a clear inflation trend interrupted by small deflation phases on 2004 and 2007. On the other hand, concerning the CSK time window the cGNSS data are available for all the 4 stations and also in this case the time series show a very good agreement with the InSAR ones for both ascending and descending tracks.

**Fig. 11:**
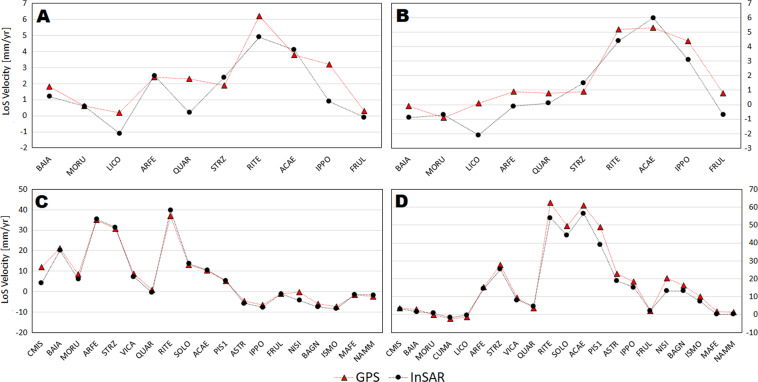
Comparison between LoS velocity retrieved from InSAR and cGNSS data for Envisat ascending (**A**) and descending (**B**) and CSK ascending (**C**) and descending (**D**) space missions.

Additionally, the LoS velocities from InSAR and cGNSS at all the available stations were also compared (Fig. 11). As shown in the time series, the displacement rate is not constant at Campi Flegrei, therefore the mean velocities may be not representative of the alternating phases of ground displacement deceleration and acceleration. However, considering the time intervals covered by Envisat (2003–2010) and CSK (2011–2021) missions, it is a reliable approximation since the caldera is undergoing a continuous uplift with an increasing rate starting in 2011, i.e., just after the end of Envisat and the beginning of CSK datasets. Indeed, the agreement between InSAR and cGNSS measurements is very good with RMSE values spanning from about 1 and 4 mm/year (Table 4).

**Table 4:** RMSE between InSAR and cGNSS measurements.

Mission	Track	RMSE [mm/yr]
Envisat	Ascending	1.18
Envisat	Descending	1.11
CSK	Ascending	2.35
CSK	Descending	3.93

## Data Availability

All the SAR acquisitions were processed by means the commercial software GAMMA© developed by the Swiss corporation GAMMA Remote Sensing AG (see https://www.gamma-rs.ch/). Annual updating of CSK InSAR products can be accessed via the INGV Iridium portal or upon request to the corresponding author.
